# Twenty years and counting: epidemiology of an outbreak of isoniazid-resistant tuberculosis in England and Wales, 1995 to 2014

**DOI:** 10.2807/1560-7917.ES.2017.22.8.30467

**Published:** 2017-02-23

**Authors:** Catherine M Smith, Suzan C M Trienekens, Charlotte Anderson, Maeve K Lalor, Tim Brown, Alistair Story, Hannah Fry, Andrew C Hayward, Helen Maguire

**Affiliations:** 1 Farr Institute of Health Informatics Research, Department of Infectious Disease Informatics, University College London, London, United Kingdom; 2 These authors contributed equally to this work; 3 Field Epidemiology Service, Liverpool, United Kingdom; 4 Field Epidemiology Training Programme, Public Health England, London, United Kingdom; 5 European Programme for Intervention Epidemiology Training, European Centre for Disease Prevention and Control, Stockholm, Sweden; 6 Field Epidemiology Service – South East and London, Public Health England, London, United Kingdom; 7 Public Health England TB Section, Centre for Infectious Disease Surveillance and Control, Colindale, London, United Kingdom; 8 Public Health England National Mycobacterium Reference Laboratory, Whitechapel, London, United Kingdom; 9 Find and Treat, University College Hospitals NHS Foundation Trust, London, United Kingdom; 10 Centre for Advanced Spatial Analysis, University College London, London, United Kingdom; 11 Research Department Infection and Population Health, Centre for Infectious Disease Epidemiology, University College London, London, United Kingdom

**Keywords:** tuberculosis, antimicrobial resistance, outbreaks, typing, geographic information system – GIS, social risk factors

## Abstract

An outbreak of isoniazid-resistant tuberculosis first identified in London has now been ongoing for 20 years, making it the largest drug-resistant outbreak of tuberculosis documented to date worldwide. We identified culture-confirmed cases with indistinguishable molecular strain types and extracted demographic, clinical, microbiological and social risk factor data from surveillance systems. We summarised changes over time and used kernel-density estimation and k-function analysis to assess geographic clustering. From 1995 to 2014, 508 cases were reported, with a declining trend in recent years. Overall, 70% were male (n = 360), 60% born in the United Kingdom (n = 306), 39% white (n = 199), and 26% black Caribbean (n = 134). Median age increased from 25 years in the first 5 years to 42 in the last 5. Approximately two thirds of cases reported social risk factors: 45% drug use (n = 227), 37% prison link (n = 189), 25% homelessness (n = 125) and 13% alcohol dependence (n = 64). Treatment was completed at 12 months by 52% of cases (n = 206), and was significantly lower for those with social risk factors (p < 0.05), but increased over time for all patients (p < 0.05). The outbreak remained focused in north London throughout. Control of this outbreak requires continued efforts to prevent and treat further active cases through targeted screening and enhanced case management.

## Introduction

Incidence rates of tuberculosis have fallen in many European countries in recent years, but were increasing until 2009 in England and Wales, and have since remained relatively high [1]. In 2014, 6,520 cases were reported in England (12/100,000 inhabitants) and 115 in Wales (4/100,000). The highest incidence rate (30/100,000) was reported in London, where 39% of cases in England resided [2]. Multidrug-resistant (MDR) disease poses a particular threat to tuberculosis control as it cannot be managed using standard treatment regimens. Resistance to a single first-line drug is a precursor for development of MDR-tuberculosis, and isoniazid resistance is the most commonly identified form of resistance worldwide [3].

In England and Wales, 6–7% of cases with drug-susceptibility results are resistant to isoniazid [2], and in 2013, 7% of isoniazid-resistant tuberculosis cases in England had a strain type known to be associated with an ongoing outbreak [4]. This outbreak was first identified in 2000 at a hospital in north London where three young men were diagnosed with an identical strain type of the Euro-American lineage within a week. Retrospective strain typing of isolates available at the time identified a further 15 cases, with the first case dating back to 1995 [5]. Cases have since been ascertained prospectively, and the outbreak now spans 20 years [6].

Epidemiological characteristics of this ongoing outbreak were last described for cases up to 2006 [7]. These cases have previously been shown to include a high proportion of young males, particularly of white or black Caribbean ethnicity, who were born in the United Kingdom (UK) and lived in north London. Cases were also significantly more likely to present with social risk factors including imprisonment, unemployment, drug use or sex work [5,7,8]. An Outbreak Control Committee (OCC) established in 2000 recommended action on interagency working, improved identification and management of cases including use of directly observed therapy (DOT) and contact tracing, and improved control in prisons [9].

As cases continue to be reported 20 years since the first identified case, this cluster now represents one of the largest documented outbreaks worldwide of drug-resistant tuberculosis. In this study, we aimed to describe the evolution of the outbreak in time and space and discuss implications for future tuberculosis control.

## Methods

### Outbreak case definition and data sources

Cases were defined as individuals diagnosed from 1995 to 2014 in England and Wales with a *Mycobacterium tuberculosis* isolate that was indistinguishable from the outbreak strain. Following identification of the outbreak in January 2000, cases were ascertained prospectively by strain typing of all isoniazid-resistant isolates from patients resident in, or with known epidemiological links to, London. Prior to 2000, cases were ascertained retrospectively through review of microbiological databases and strain typing of identified isolates [7]. From 2010 onwards, strain typing was conducted on all tuberculosis isolates in England and Wales, regardless of links to London.

The outbreak strain was initially characterised using restriction fragment length polymorphism (RFLP) analysis and before 2000, the isolates selected for typing were those of isoniazid monoresistant organisms cultured at four laboratories serving the area where first cases were reported. After 2000 all such strains across London were RFLP-typed, and from 2006 onwards, due to a change in routine practice, 24-locus mycobacterial interspersed repetitive sequence variable-number tandem repeat (MIRU-VNTR) typing was used to identify the corresponding strain [7]. Strain typing was conducted at the Health Protection Agency National Mycobacterium Reference Laboratory.

We extracted information on outbreak-related cases from multiple data sources. Demographic, clinical, microbiological and treatment outcome data were provided by a bespoke outbreak database, and from the electronic surveillance systems for London (the London Tuberculosis Register, LTBR) and the rest of England and Wales (the Enhanced Tuberculosis Surveillance System, ETS). Information on social risk factors (drug use, link to prisons, including patients who were in prison at time of diagnosis, homelessness, alcohol dependence and mental health concerns) was collected initially in the bespoke outbreak database, and from 2009 in surveillance systems.

We also extracted information on outbreak cases from data collected by Find and Treat, a pan-London tuberculosis outreach service [10]. This service aims to identify cases of tuberculosis in hard-to-reach populations, typically those with social risk factors, and support them to complete treatment. We identified outbreak-related cases who had been screened and managed by the service, and used data to supplement information on social risk factors.

Databases were combined on the basis of unique identifiers, patient names and dates of birth. Patients with multiple episodes of tuberculosis with the outbreak strain were identified, but only the first period was included in analyses.

### Epidemiological analysis

We plotted annual numbers of outbreak cases as an epidemic curve. We described demographic, clinical and microbiological characteristics of all cases in counts and proportions, and used the chi-squared test for trend to identify changes over time. For social risk factors, we calculated overall proportions and identified changes over time by plotting proportions of cases reporting risk factors by year.

We identified treatment outcomes at 12 months for non-MDR cases who were notified between 2002 and 2013. MDR cases were excluded from this analysis because their planned treatment regime exceeds 12 months; cases notified before 2002 were excluded as they did not have a date of treatment outcome recorded, and outcomes had not yet been collected for cases after 2013. We tested for changes in proportions of cases completing treatment over time and used the chi-squared test to compare proportions of cases with and without social risk factors who completed treatment. Final known outcomes were also defined, and included cases notified before 2002, MDR cases notified before 2013, and incorporated 12- or 24-month treatment follow-up where appropriate.

We used Find and Treat data to identify the proportion of cases notified in London who had been screened by the service (cases from 2005 onwards), and referred for case management (cases from 2007 onwards). We calculated the proportions of patients referred to Find and Treat who had social risk factors, and used chi-squared tests to compare the rates of treatment completion in patients referred to Find and Treat with those who were not.

### Spatial analysis

We determined case locations using geocoded residential postcodes where available. Prison or clinic postcodes were used where relevant for patients diagnosed while in prison or with no fixed abode. We plotted numbers of cases nationally by region and calculated incidence rates by London borough using population data from the 2001 census. We visualised the spatio-temporal progression of the outbreak within London through a series of smoothed-incidence maps. Each map displays the spatial intensity of case locations in a 5-year period during the outbreak, generated through kernel-density estimation [11].

We further explored the spatial point pattern of cases in London through k-function analysis. The k-function is a method for detecting spatial clustering and is defined as the expected number of cases within a given distance from an arbitrary case location [12]. First, we tested the hypothesis that the points were completely spatially random by comparing the k-function of the observed point locations with the function generated by 99 simulated point patterns. We then tested the hypothesis that the locations of cases in the first 10 years of the outbreak were part of the same spatial distribution as those in the second decade by calculating their cross k-function. This is the number of points from one distribution within a range of distances of a typical point from the other distribution [11]. The observed cross k-function was compared with the functions defined by 99 simulations based on random re-labelling of the joint spatial distribution of points to the two time periods. If the observed function lay within the upper and lower bounds of these limits, this would be consistent with the null hypothesis that the points were part of a common spatial distribution.

Data management, validation and analysis were performed using R 3.1 and Stata 13.0. The R package *spatstat* was used for kernel-density and k-function analyses [13].

## Results

**Figure 1:**
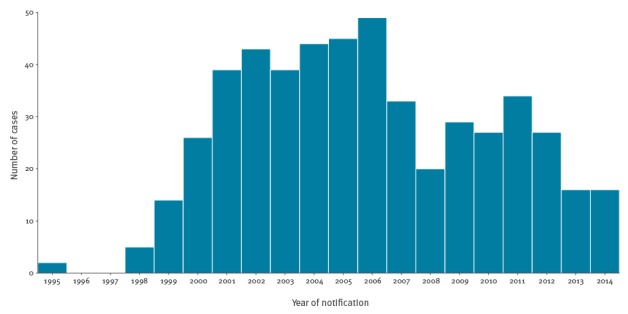
Number of cases in the isoniazid-resistant tuberculosis outbreak by year, England and Wales, 1995–2014 (n = 508)

From 1995 to 2014, 508 cases with the isoniazid-resistant tuberculosis outbreak strain were identified. The epidemic curve (Figure 1) shows that, after initial ascertainment of the outbreak in 2000, the number of cases rose steeply, reaching a peak of 49 in 2006. After a subsequent decrease in numbers, there appears to have been a second peak in 2011, followed by another decline in cases.

### Characteristics of cases

Table 1 presents the demographic characteristics of cases in this outbreak by 5-year period. The majority of cases (71%) were male; of white (39%), black Caribbean (26%) or black African (13%) ethnicity; and born in the UK (60%). There were no significant changes in proportions of these characteristics over time (chi-squared trend p = 0.97, 0.39, and 0.28 for sex, ethnicity and place of birth respectively). The median age of cases increased from 25 years (range 6–71, interquartile range (IQR) 21–28) in 1995–1999 to 42 in 2010–2014 (range 12–79, IQR 31–49); and there was a significant increase in the proportion of cases aged 45–64 years over the outbreak (chi-squared trend: p < 0.001).

**Table 1:** Demographic characteristics of cases in the isoniazid-resistant tuberculosis outbreak, England and Wales, 1995–2014

	1995–1999 n (%)	2000–2004 n (%)	2005–2009 n (%)	2010–2014 n (%)	All n (% of total)
All cases	21	191	176	120	508
**Sex**					
Male	13 (61.9)	139 (72.8)	122 (69.3)	86 (71.7)	360 (70.9)
Female	8 (38.1)	52 (27.2)	54 (30.7)	34 (28.3)	148 (29.1)
**Age (years)**					
Median	25	35	36	42	36
< 15	1 (4.8)	1 (0.5)	5 (2.8)	2 (1.7)	9 (1.8)
15–24	9 (42.9)	31 (16.2)	22 (12.5)	8 (6.7)	70 (13.8)
25–34	8 (38.1)	60 (31.4)	53 (30.1)	29 (24.2)	150 (29.5)
35–44	1 (4.8)	58 (30.4)	55 (31.3)	31 (25.8)	145 (28.5)
45–64	1 (4.8)	34 (17.8)	35 (19.9)	46 (38.3)	116 (22.8)
> 65	1 (4.8)	7 (3.7)	6 (3.4)	4 (3.3)	18 (3.5)
**Ethnic group**					
White	8 (38.1)	59 (30.9)	73 (41.5)	59 (49.2)	199 (39.2)
Black Caribbean	4 (19.0)	59 (30.9)	49 (27.8)	22 (18.3)	134 (26.4)
Black African	3 (14.3)	34 (17.8)	20 (11.4)	10 (8.3)	67 (13.2)
Indian	2 (9.5)	7 (3.7)	6 (3.4)	7 (5.8)	22 (4.3)
Black other	1 (4.8)	4 (2.1)	8 (4.5)	5 (4.2)	18 (3.5)
Other	1 (4.8)	18 (9.4)	14 (8.0)	15 (12.5)	43 (8.4)
Unknown	2 (9.5)	10 (5.2)	6 (3.4)	2 (1.7)	20 (3.9)
**UK-born**					
Yes	15 (71.4)	95 (49.7)	114 (64.8)	82 (68.3)	306 (60.2)
No	4 (19.0)	82 (42.9)	51 (29.0)	35 (29.2)	172 (33.9)
Unknown	2 (9.5)	14 (7.3)	11 (6.3)	3 (2.5)	30 (5.9)
**Country/area of birth if not UK-born**					
Sub-Saharan Africa^a^	1 (4.8)	24 (12.6)	20 (11.4)	8 (6.7)	53 (10.4)
Jamaica	0 (0)	17 (8.9)	10 (5.7)	5 (4.2)	32 (6.3)
Ireland	1 (4.8)	14 (7.3)	2 (1.1)	6 (5.0)	23 (4.5)
Indian Subcontinent^b^	1 (4.8)	4 (2.1)	3 (1.7)	6 (5.0)	14 (2.8)
Other	0 (0)	20 (10.5)	15 (8.5)	10 (8.3)	45 (8.9)
Unknown	3 (14.3)	17 (8.9)	12 (6.8)	3 (2.5)	35 (6.9)

UK: United Kingdom.

^a^ Includes cases born in Angola, Congo, Eritrea, Ethiopia, Gambia, Ghana, Kenya, Liberia, Mauritania, Nigeria, Sierra Leone, Somalia, Tanzania, Uganda, Zambia and Zimbabwe.

^b^ Includes cases born in India, Bangladesh, Pakistan and Sri Lanka.

Most cases (85%) had pulmonary tuberculosis, and this proportion did not change over time (chi-squared trend: p = 0.83). All cases had isoniazid-resistant disease; there were 14 cases of MDR-tuberculosis (3%), of which nine were MDR at their initial drug resistance test, and five were initially isoniazid-resistant but subsequently acquired resistance to rifampicin. One MDR case additionally developed pyrazinamide resistance. Twenty-four patients were diagnosed with this strain on more than one occasion, half of whom had initially completed treatment. The longest interval between diagnoses was 14 years; median 3.5 years.

**Figure 2:**
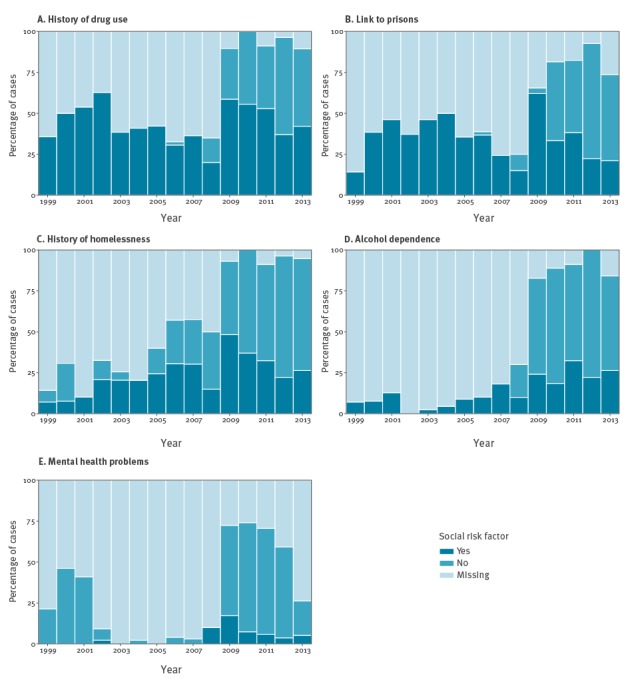
Percentage of cases in the isoniazid-resistant tuberculosis outbreak by year and social risk factor, England and Wales, 1999–2014

One or more social risk factors were reported for 308 (61%) cases. History of drug use (227, 45%), links to prisons (189, 38%), and homelessness (125, 25%) were most frequently reported. Alcohol dependence (64, 13%) and mental health concerns (13, 3%) were reported less often. For 108 cases, two risk factors were reported (21%); for 66 cases, three risk factors (13%) and for 23 cases, four risk factors (5%). Figure 2 displays the proportion of cases with each social risk factor by year. This demonstrates the continued importance of prisons and drug use over the duration of the outbreak; as well as the change in data-collection methods in 2009, which resulted in an increased proportion of cases reported with presence or absence of risk factors and decreased proportion with missing data. Prior to this, reports were commonly made only if a risk factor was present and left missing if absent.

### Treatment outcomes and Find and Treat

**Figure 3:**
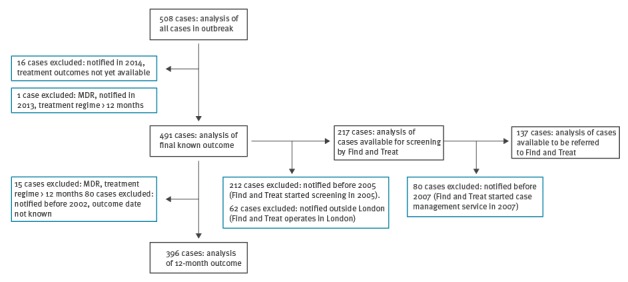
Eligibility criteria for cases included in analyses of treatment outcomes and Find and Treat data, isoniazid-resistant tuberculosis outbreak, England and Wales, 1995–2014

Figure 3 defines the eligibility criteria for including cases in these analyses.

MDR: multidrug-resistant.

Black boxes show analyses, blue boxes show exclusions.

Treatment was completed by 206 (52%) of 396 eligible patients at 12 months, with a significant increase in this proportion during the outbreak (p < 0.05). Cases with at least one social risk factor had a significantly lower percentage of treatment completion at 12 months than those with none or missing information on social risk factors (42% and 67% respectively, chi-squared test: p < 0·05). Treatment completion was lowest for those with a history of homelessness (n = 42/125, 39%), links to prisons (n = 58/189, 39%), or a history of drug use (n = 72/227, 52%). At final known outcome, 372 (76%) of 491 eligible cases completed treatment, and those with at least one social risk factor also had a lower percentage of treatment completion (71% vs 82% for those with none or missing information on social risk factors, chi-squared test: p = 0·006). Twenty cases were reported to have died (4%). Tuberculosis is known to have contributed to the deaths of three patients at final known outcome, was incidental for seven, and has an unknown link to the deaths of the remaining 10 patients. Reasons for failing to complete treatment are shown in Table 2; 8.6% and 8.8% of cases had been lost to follow-up at 12 months and final known outcome respectively.

**Table 2:** Treatment outcomes of cases in the isoniazid-resistant tuberculosis outbreak, England and Wales, 2002–2013 (12-month outcome) and 1995–2013 (final known outcome)

Outcome	12-month outcome (cases 2002–2013)^a^ n (%)	Final known outcome (cases 1995–2013)^b^ n (%)
Completed	206 (52.0)	372 (75.8)
Still on treatment	66 (16.7)	11 (2.2)
Lost to follow-up	34 (8.6)	43 (8.8)
Died	12 (3.0)	20 (4.1)
Transferred out	10 (2.5)	17 (3.5)
Unknown / Not complete – unknown reason	68 (17.2)	28 (5.7)
**Total**	**396**	**491**

^a^ Excluding all multidrug-resistant cases.

^b^ Excluding multidrug-resistant case notified in 2013.

The Find and Treat screening programme aims to identify cases of tuberculosis in ‘hard to reach’ populations and has been operating in London since 2005. During this period (2005–2013), it screened 11.5% (25/217) of the individuals which were subsequently found to be part of the outbreak. Since 2007, Find and Treat has also operated a case management service, and one quarter (35/137) of outbreak patients notified in London in this time period have been referred to the service. The majority of these patients had a history of homelessness (n = 30/35); drug use (n = 29/35) and links to prisons (n = 21/35). These patients were significantly less likely to have completed treatment at 12 months (15/35) compared with those who were not managed within the service (72/102, chi-squared p = 0.006). However, treatment completion at final known outcome was not significantly different between the two groups (26/35 vs 87/102; chi-squared test: p = 0.22).

### Spatial analysis

**Figure 4:**
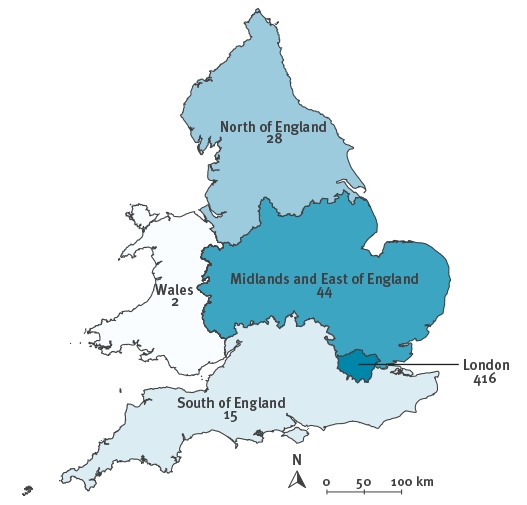
Numbers of cases in the isoniazid-resistant outbreak in England (by Public Health England Region) and Wales, 1995−2014 (n=505)

All cases were successfully geocoded to locations in England and Wales, with the exception of which three had no location data. The majority of these cases (416, 82%) lived in London; while the Midlands and East of England (44, 9%) reported the most cases of other regions (Figure 4). Within London, most cases were reported in north-east and north central areas, with the highest rates in the boroughs of Hackney and Haringey (45 and 34 cases per 100,000 population respectively), compared with 6 per 100,000 for the whole of London.

Total number of cases is 505 because three cases had no geographical information. Contains Ordnance Survey data, Crown copyright and database right 2014.

**Figure 5:**
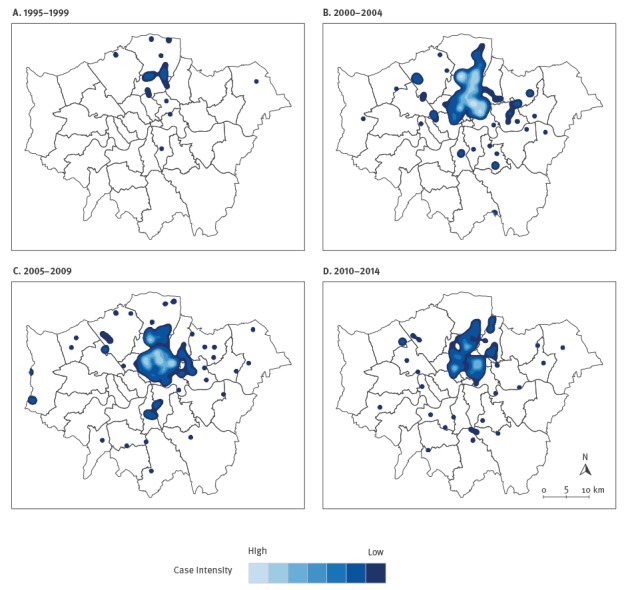
Smoothed incidence maps of cases in the isoniazid-resistant tuberculosis outbreak in London, by 5-year time period, 1995–2014

The smoothed incidence maps (Figure 5) show that the outbreak has remained largely concentrated in north London, with the highest spatial intensity of cases located in this region in all four time periods.

Spatial intensity determined using kernel-density estimation, bandwidth 597 m. Contains Ordnance Survey data, Crown copyright and database right 2014.

**Figure 6:**
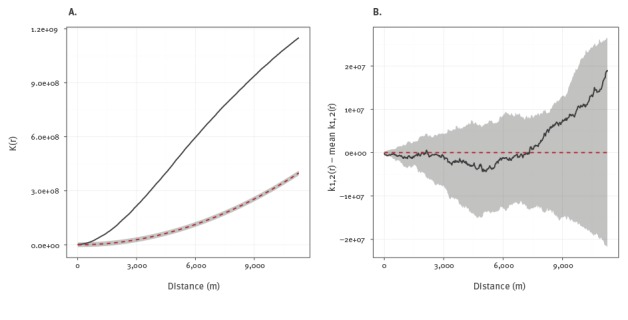
K-function analysis of spatial clustering in isoniazid-resistant tuberculosis outbreak in London, 1995–2014; A: K-function test of complete spatial randomness; B: Cross k-function comparing first and second 10 years of outbreak

The k-function of the observed point locations (Figure 6A) lies clearly outside the simulation envelope representing randomly generated point patterns. This demonstrates that the data show spatial clustering above what would be expected by complete spatial randomness. The cross k-function (Figure 6B) compares the spatial distribution of the cases in the first and second 10 years of the outbreak. The k-function of the observed data lies within the simulation envelope generated through random labelling of cases to different time periods. There was therefore no evidence that the spatial distribution of cases in London changed significantly during the outbreak.

Simulation envelopes shaded in grey.

## Discussion

This isoniazid-resistant tuberculosis outbreak has now been ongoing for 20 years, despite a recent decline in incidence. It has had a consistent focus in north London, particularly among socially marginalised populations. Links to prisons, drug use, and a history of homelessness are important risk factors, and failure of these groups to complete treatment is likely to have perpetuated the outbreak.

Major impacts of this outbreak have included tuberculosis disease in over 500 individuals, at least three linked deaths, and reinfection or relapse in 24 cases. Multidrug-resistance has emerged in this strain, and appears to have been transmitted between cases, as nine patients presented with an initial drug resistance test that was MDR. There are also potentially thousands of further individuals who have undetected infections, given that the lifetime risk of developing active disease following infection is estimated at 10% [14]. Furthermore, this outbreak has contributed considerable economic costs to health and social care services: Management of an uncomplicated case of tuberculosis is estimated to cost GBP 5,000 (approximately EUR 5,790), while drug-resistant cases can cost more than 10 times this amount, before taking into account use of additional resources associated with outbreak investigations such as contact tracing and outbreak control team meetings [15].

The outbreak has proved particularly challenging to control despite great efforts overseen by the dedicated OCC established in 2000. It has consistently been associated with ‘hard to reach’ populations including those with a history of drug use, homelessness or imprisonment. Recommendations implemented by the OCC which attempted to target these groups included extension of contact tracing beyond household contacts to close social contacts, promulgation of advice relating to specific drug regimens for treatment, and expanded use of DOT [9]. The OCC met regularly and reviewed progress and implementation, with improvements seen in numbers of contacts traced per case and treatment completion, but the decline in cases was slow to occur. In more recent years, the Find and Treat mobile screening unit has contributed to control of the outbreak. Approximately one in 10 outbreak cases was screened by this service since it started operating, and it was also an effective service for managing complex patients: In this outbreak, patients managed by Find and Treat had a higher prevalence of social risk factors, but there was no significant difference in final treatment completion rates in these patients compared with others in the outbreak.

Our analysis provides some insights into the natural history of this outbreak and how it has progressed. In spite of its long duration, the outbreak has remained fairly circumscribed in north London, and characteristics of populations affected have remained relatively stable, although the age of the patients at notification did increase over time. The smoothed incidence maps and k-function analyses demonstrate distinct spatial clustering which persisted in the same region of north London throughout. These observations are consistent with intensive transmission among a social cohort approximately 20 years ago, whose infections have gradually progressed to active disease. If a great deal of ongoing transmission had been occurring outwith these groups, it would be expected that cases would have become more widely disseminated with smaller clusters arising in dispersed geographic areas. Detailed genetic analyses of a selection of outbreak isolates found little or no associated fitness cost and the presence of specific deletions that could be a peculiar feature of the strains and help explain their persistence over the very many years [16].The epidemic curve had a two-wave pattern, with an initial peak which may represent cases whose infections rapidly progressed to disease, and a later peak potentially driven by those presenting with symptoms following a longer period of latency. Alternatively, the second wave of cases may have resulted from a second period of intensive transmission. Whole genome sequencing of isolates could be used to investigate these hypotheses by constructing a phylogenetic tree that identifies likely chains of transmission [17].

This analysis represents the largest documented outbreak of drug-resistant tuberculosis to date. Previous outbreaks of comparable size have been reported in New York City [18-20] and South Africa [21,22]. Both incidents were linked to nosocomial transmission among HIV-positive patients, which have not been important factors in this outbreak [7]. There are more commonalities between this outbreak and one that occurred in Stockholm, Sweden between 1996 and 2005, comprising 96 cases [23,24]. The Stockholm outbreak was also characterised by confinement of cases to a distinct demographic group in a small geographic area, resistance to isoniazid, and had an epidemic curve with a two-wave pattern. This indicates the importance of community transmission of tuberculosis within European cities, and the need to focus control measures on affected groups.

Our results therefore have implications for future control of this outbreak and for control of tuberculosis more widely. We recommend targeted screening of high-risk individuals (for example in prisons) to prevent further active cases, and enhanced case management to support patients to complete treatment. Continued support of the Find and Treat tuberculosis outreach service, which provides a cost-effective approach to case finding [10] and has successfully identified and managed complex patients in this outbreak, should help to achieve this. We additionally recommend following National Institute for Health and Care Excellence guidance for tackling tuberculosis among hard-to-reach groups [15]. This includes standardised risk assessment for all tuberculosis patients; better recording and monitoring of contact tracing, and expanded use of DOT, which is used infrequently in London compared with other parts of the world [25]. In the wider context of tuberculosis outbreak control, we recommend regular reviews of the epidemiology and spatial distribution of tuberculosis clusters linked by molecular strain typing. This will enable better understanding of the important factors associated with transmission, tracking the extent of spatial dispersion of outbreak strains, and improved targeting of control measures.

A strength of this study is that we combined data from multiple sources including two surveillance systems, a bespoke outbreak database, and data from the tuberculosis outreach service, Find and Treat. This enabled us to describe all reported cases that have been associated with the outbreak, and ensured best possible completeness of variables. However, there were also limitations with these data: We are unlikely to have ascertained all cases affected in the outbreak because we used only culture-confirmed cases, excluding those without appropriate strain-typing information regardless of epidemiological links. This also means that we are likely to have overestimated the prevalence of pulmonary disease in this outbreak. The number of cases that occurred before 2000 is likely to have been underestimated as cases were ascertained retrospectively, and before 2010 cases notified outside London were not typed unless there were known epidemiological links to the outbreak. Owing to the change in routine microbiological testing procedures from RFLP to MIRU-VNTR typing, the case definition for this outbreak also changed. It is therefore possible that these cases are not part of the same outbreak. However, all cases were isoniazid-resistant and epidemiological characteristics of cases did not change significantly following the change in strain-typing methodology. This suggests that the updated case definition was appropriate, but this is being confirmed through whole genome sequencing of isolates defined through the two different methods.

Another limitation of this analysis is that we were unable to assess the importance of risk factors which are not included in routine surveillance. These include commercial sex work and unemployment, which have previously been found to be linked to this outbreak, and HIV status, which is not thought to be an important factor [7]. Estimates for prevalence of social risk factors also represent minimum likely values, owing to non-ascertainment, non-disclosure and inconsistent definitions, particularly before 2009 when these fields were introduced to routine surveillance systems. We therefore did not assess the relative importance of risk factors in this outbreak through a formal case control study. However, surveillance data shows that the proportion of all London tuberculosis cases reported between 2009 and 2014 who had one or more social risk factor was substantially lower than for cases in this outbreak reported over the same period (ca 10% and 40% respectively) [6]. The shift towards older age groups observed here has also not been observed in London cases more widely, and, contrary to the pattern in this outbreak, highest overall rates within London have been in north-west areas [6]. It is therefore likely that the characteristics identified here are specific to this outbreak and do not merely reflect the epidemiology of tuberculosis patients as a whole.

Finally, there were limitations to the statistical and spatial methods used in this analysis. We used the chi-squared test for trend to identify changes in patient characteristics over time. An assumption of this test is that the proportions did not, for example, increase and then decrease as the outbreak progressed. We checked for this possibility by plotting these characteristics as a function of time. Spatial analyses were based on point locations of cases stratified by 5- and 10-year time periods. This provides an incomplete picture of the true spatial distribution of the outbreak and could have masked intra-period changes in distributions. However, the smoothed incidence maps demonstrate a clear and persistent focal point the north of the city, so it is unlikely that these factors have had a substantial impact on the conclusions.

Tuberculosis in Europe is increasingly a problem that is concentrated in large cities [26]. This study demonstrates that outbreaks in cities, even in low incidence countries, can persist for many years through community transmission. Resolving this outbreak of drug-resistant disease, and prevention of future outbreaks, will therefore be a key factor in strengthening tuberculosis control in Europe. As recognised by the recent Collaborative Tuberculosis Strategy for England, this will require that best practice in clinical care, social support and public health are brought together [27].
